# A new static visual field test algorithm: the Ambient Interactive ZEST (AIZE)

**DOI:** 10.1038/s41598-023-42266-z

**Published:** 2023-09-11

**Authors:** Hiroki Nomoto, Chota Matsumoto, Sachiko Okuyama, Shinji Kimura, Satoshi Inoue, Kenzo Yamanaka, Shunji Kusaka

**Affiliations:** 1 Department of Ophthalmology, Faculty of Medicine, Kindai University, Osaka-sayama, Japan; 2 CREWT Medical Systems Inc., Tokyo, Japan

**Keywords:** Glaucoma, Diagnosis

## Abstract

Visual field (VF) test is one of the most vital tests in the diagnosis of glaucoma and to monitor the disease worsening. In the past couple of decades, the standard automated perimetry (SAP) test takes a major role in VF test for glaucoma patients. The SAP has been demanded to finish a test in short time without sacrificing accuracy. In this study, we developed and evaluated the performance of a new perimetric algorithm (ambient interactive zippy estimation by sequential testing (ZEST): AIZE) by computer simulation. AIZE is a modification of the ZEST procedure that utilizes the spatial information (weighted likelihood: WL) of neighboring test locations, which varies from the distance to the tested location, to estimate a visual threshold. Ten glaucomatous and 10 normal empirical visual field (VF) test results were simulated with five error conditions [(3% false positives (FP), 3% false negatives (FN)), (9% FP, 9% FN), (15% FP, 15% FN), (3% FP, 15% FN), (15% FP, 3% FN)]. The total number of test presentations and the root mean square error (RMSE) of the estimated visual sensitivities were compared among AIZE, the non-weighted test (WL = 0) and the fixed-weighted test (WL = 0.33). In both glaucomatous (G) and normal (N) VFs, the fixed-weighted test had the lowest number of test presentations (median G 256, N 139), followed by the AIZE (G 285, N 174) and the non-weighted test (G 303, N 195). The RMSE of the fixed-weighted test was lower (median 1.7 dB) than that of the AIZE (1.9 dB) and the non-weighted test (1.9 dB) for normal VFs, whereas the AIZE had a lower RMSE (3.2 dB) than the fixed-weighted test (4.5 dB) and the non-weighted test (4.0 dB) for glaucomatous VFs. Simulation results showed that AIZE had fewer test presentations than the non-weighted test strategy without affecting the accuracy for glaucomatous VFs. The AIZE is a useful time saving test algorithm in clinical settings.

## Introduction

The standard automated perimetry (SAP) test is the most common procedure for clinicians and researchers to detect and monitor glaucomatous visual field (VF) changes. The SAP uses the Goldmann size III stimulus target to measure visual sensitivity at various locations across the central 30 degrees. In the 1980s, initial SAP threshold algorithms, such as the full-threshold algorithm of the Humphry Field Analyzer (HFA: Carl Zeiss Meditec, Inc., Dublin, CA, USA), took 12–20 min per eye to complete in glaucoma patients^1–3^. It was sometimes too long for patients to endure, leading to poor test results^4–6^.

The Swedish Interactive Threshold Algorithm (SITA) strategies became commercially available for the HFA in the early 1990s. The SITA uses a Bayesian estimation of threshold values for each location and the response window method to reduce test time^7^. The SITA-Standard enabled shortening of the test time by approximately 50% compared to the full-threshold algorithm^3^. Currently, SITA strategies are the standard for measuring VF sensitivity both in clinical practice and research.

The perimetric threshold test requires not only higher speeds but also higher accuracy. An idea that uses information from neighboring test points to estimate VF sensitivities has been proposed to achieve faster and more accurate VF tests. In the 1970s, Heijl et al.^8^ devised an innovative test algorithm that estimates sensitivities at four primary locations and then spreads the test locations in sequential order. The SITA also uses spatial information to estimate visual sensitivity;^9^ however, the details have not been revealed to the public. In recent years, Chong et al.^10^ and Rubinstein et al.^11^ introduced a new algorithm that uses spatial information to measure the VF. They concluded that their algorithms have the potential to reduce the test time without affecting accuracy.

We developed a new VF test strategy, the Ambient Interactive ZEST (AIZE), which updates estimations of VF sensitivity at the tested location as well as at neighboring locations simultaneously by using spatial information for every stimulus presentation in the process of estimating the VF sensitivities. In this study, we verified the efficiency of using spatial information to reduce the test time without sacrificing accuracy with computer simulations.

## Results

**Figure 1:**
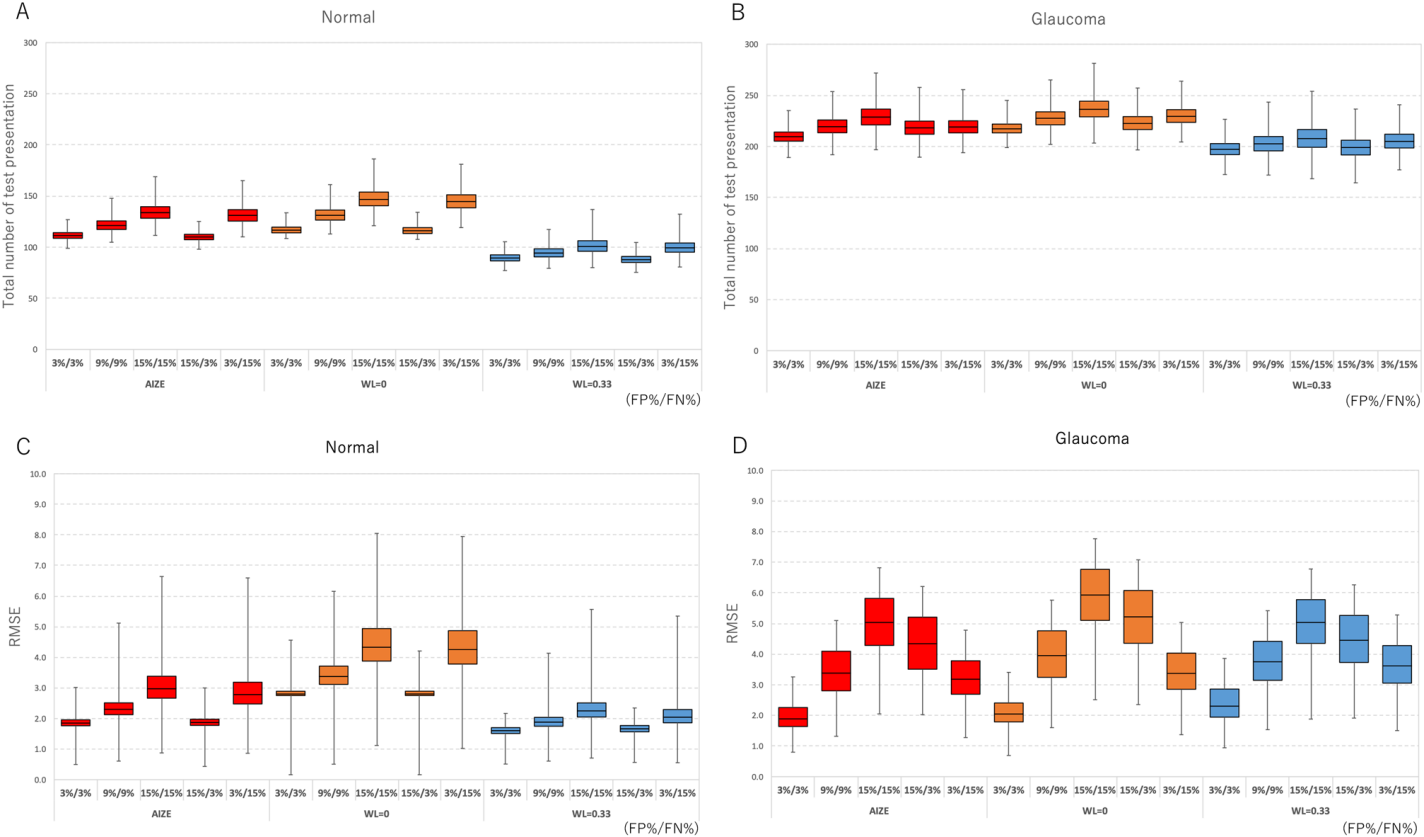
The total number of test presentation with AIZE, the non-weighted and the fixed-weighted tests under 5 error conditions in normal (**A**) and glaucoma (**B**). The RMSE with AIZE, the non-weighted and the fixed-weighted tests under 5 error conditions in normal (**C**) and glaucoma (**D**). (FP: false positive rate, FN: false negative rate).

For normal VFs, the total number of test presentations for the AIZE (median of the five error conditions: 121, interquartile range (IQR): 111, 124) was fewer than that of the non-weighted test (median 131, IQR: 126, 134) (*p* < 0.001) and larger than that of the fixed-weighted test (median 94, IQR: 83, 98) (*p* < 0.001) (Fig. 1A). The total number of test presentations for glaucomatous VFs was similar to that for normal VFs. The AIZE (median 219, (IQR): 203, 224) had fewer test presentations than the no-weighted test (median 228, IQR: 213, 232) (*p* < 0.001) and more test presentations than the fixed-weighted test (median 203, IQR: 183, 208) (*p* < 0.001) (Fig. 1C). The total number of test presentations with the AIZE was the second fewest among the three tests in normal and glaucomatous VFs.

The RMSE of the AIZE for normal VFs (median of the five error conditions: 2.3 dB, IQR: 1.8, 2.4) was smaller than that of the non-weighted test (median 3.4 dB, IQR: 3.2, 3.5) (*p* < 0.001) and larger than that of the fixed-weighted test (median 1.9 dB, IQR: 1.4, 2.0) (*p* = 0.002) (Fig. 1B). There was no significant difference between the AIZE and the other two tests for normal VFs, whereas for glaucomatous VFs, the smallest RMSE was achieved by the AIZE (median 3.4 dB, IQR: 2.6, 3.8), followed by the non-weighted test (median 4.0 dB, IQR: 3.3, 4.3) (*p* = 0.002) and the fixed-weighted test (median 3.8 dB, IQR: 2.8, 4.3) (*p* = 0.003) (Fig. 1D).

**Figure 2:**
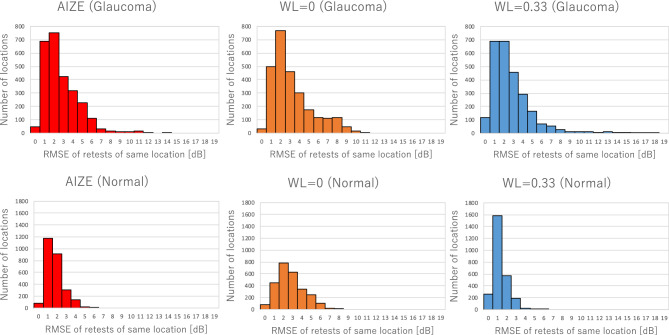
Test–retest variability AIZE, the non-weighted test (WL = 0) and the fixed-weighted test (WL = 0.33). Number of test locations were classified by the RMSE value at the same location (upper: glaucoma VF). Median value of the RMSE with AIZE, the non-weighted test and the fixed-weighted test are 2.7 dB, 3.0 dB and 2.7 dB respectively (lower: normal VF). Median value of the RMSE with AIZE, the non-weighted test and the fixed-weighted test are 2.0 dB, 3.0 dB and 1.6 dB respectively.

Figure 2 shows a histogram of test–retest variability with the three tests at the same test location. The median RMSE of the AIZE was 2.0 dB (IQR: 1.5, 2.6), which was significantly smaller than that of the non-weighted test (median 3.0 dB, IQR: 2.1, 4.1) (*p* < 0.001) and larger than that of the fixed-weighted test (median 1.6 dB, IQR: 1.2, 2.1) (*p* < 0.001) for normal VFs (Fig. 2 lower). There were no significant differences between the RMSE of the AIZE (median 2.7 dB, IQR: 1.9, 4.2) and that of the fixed-weighted test (median 2.7 dB, IQR: 1.8, 4.1) (*p* = 0.297); however, the AIZE had a smaller RMSE than the non-weighted test (median 3.0 dB, IQR: 2.2, 4.7) (*p* < 0.001) for glaucomatous VFs (Fig. 2 upper).

## Discussion

In this study, we introduced a newly developed visual field test algorithm AIZE and assessed the performance with five error conditions by computer simulation. AIZE uses spatial information to reduce test time without impairing accuracy and reliability. The efficacy of incorporating spatial information into the visual field test strategy was evaluated by the number of test presentation times and the RMSE between the true and estimated sensitivity thresholds.

The simulation-based results from normal VFs demonstrated that the fixed-weighted test (WL = 0.33) and AIZE had fewer presentations than the non-weighted test (WL = 0) for all error conditions (Fig. 1A). AIZE and the fixed-weighted test also had a smaller RMSE than the non-weighted test even for unreliable observations, such as the (15% FP, 15% FN) and (3% FP, 15% FN) conditions (Fig. 1C). This result indicates that using spatial information is useful for estimating normal VF sensitivities fast and accurately, as previously reported^10^. The visual sensitivities of normal VFs are generally with small differences among neighboring test locations, namely present smooth surfaces for the hill of vision. Stronger relationships among test locations make the VF smoother. Therefore, a higher WL is suitable for examining normal VFs (Fig. 2 lower).

**Figure 3:**
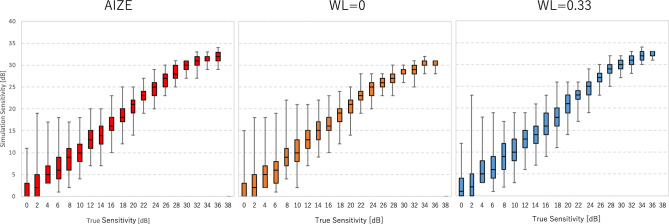
Simulation sensitivity limit of AIZE, the non-weighted test (WL = 0) and the weighted test (WL = 0.33). Boxes represent the interquartile range (25th and 75th percentiles). Vertical line indicates the 90% simulation sensitivity interval (5th and 95th percentiles).

The results for the glaucomatous VFs were similar to those of the normal VFs in terms of the total number of presentations (Fig. 1B). The accuracy of AIZE and the non-weighted tests were not significantly different; however, the fixed-weighted test was less accurate than the other two tests (Fig. 1D). VFs in the early and moderate stages of glaucoma form a localized, irregular surface for the hill of vision, such as scotomas. The depth and spatial variability of scotomas inherently influence test–retest variability^12,13^. The fixed-weighted test is not suitable for measuring a hill of vision with a rugged surface because lower (bottom of scotoma) and higher (edge of scotoma) sensitivity locations have constant strength effects against each other irrespective of distance. These relationships in regions of scotoma make any fluctuations larger and interfere with the termination of a VF test. Figure 2 shows that the AIZE has fewer test locations with a large RMSE (> 7 dB) in glaucomatous VF than the fixed-weighted and non-weighted tests. Locations with large RMSEs generally match glaucoma-damaged areas^13^. The relationship between true input sensitivity and simulation sensitivity is shown in Fig. 3. For true sensitivity of 34 dB and higher, all 3 algorithms underestimate simulated sensitivities. The non-weighted test (WL = 0) tends to underestimate in 28 dB and higher sensitivities. Whereas the weighted test (WL = 0.33) shows a larger variability of simulation sensitivities in a range of 8 to 20 dB compared to the AIZE and the non-weighted test. AIZE is a well-balanced test algorithm in terms of accuracy and speed, thus estimating glaucoma VF efficiently.

FNs and FPs are designed to measure the tendency of a subject’s responses^14–16^. FPs have a greater impact on the reliability of glaucomatous VFs than FNs^17^. Our results also showed that the RMSE was larger at high FP rates (15% FP, 3% FN) than at high FN rates (3% FP, 15% FN) (Fig. 1D). Furthermore, AIZE and the fixed-weighted test had lower RMSEs under high FP rate conditions than the non-weighted test. The FN responses depend more on the severity of disease than on a patient’s inattentiveness^18,19^. A VF from a patient with advanced-stage disease tends to yield increase FN rates and take longer to finish a test. High FN rates (15% FP, 15% FN and 3% FP, 15% FN) resulted in greater numbers of test presentations than the other error conditions, as we expected (Fig. 1B).

FN and FP response rates have a great influence on VF test result and also test time. Our algorithm set the 4 dB as a maximum standard deviation for the slope of the frequency-of-seeing curve and it is narrower than other algorithm^11^. Our 4 dB condition is unlikely to reflect unreliable responses especially in patients with moderate VF damages. Generally, unreliable responses occur at lower sensitivity locations and it is often difficult to estimate a correct sensitivity in those locations, no matter how long it takes test time. In glaucoma practice, VF test is required of detecting glaucomatous VF defects and also assessing whether a progression of the VF defects occurs or not in the shortest possible time. We presume that it could be more beneficial to evaluate a VF by reliable responses than unreliable responses for achieving a stable result and time saving. Therefore, our algorithm has more weight on reliable responses. The AIZE has already been provided in the imo perimeter^20^ and used for patients in clinical practice. Kimura et al.^21^ reported that the mean deviations of the AIZE and SITA-Standard were comparable and that the AIZE finished the VF tests significantly faster than the SITA-Standard in glaucoma patients. Similar results were found for VF defects caused by diseases in chiasmal and postchiasmal lesions^22^. Considering these results, the AIZE possesses a sufficient ability to detect VF changes and shorten the test time in practical use.

**Figure 4:**
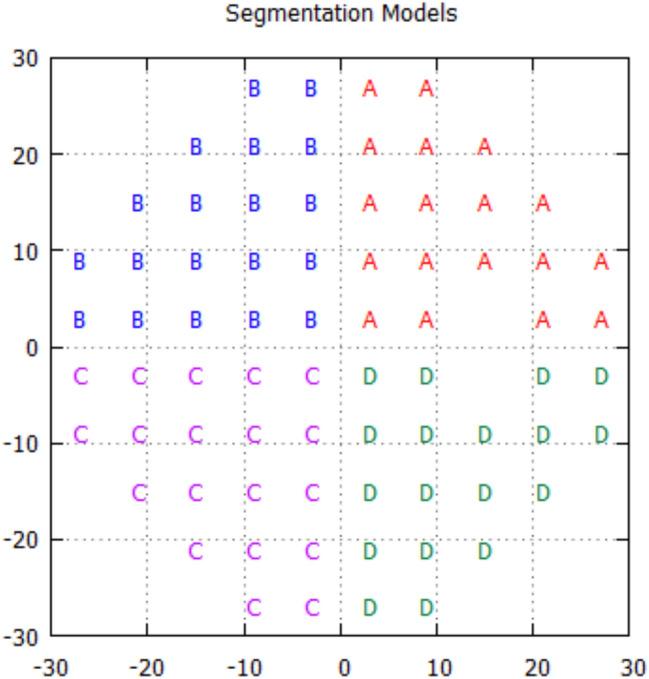
Test points were segmented into 4 quadrants (**A**–**D**).

There are two possible limitations to the AIZE. First, regarding the spatial information pattern model, numerous potential spatial pattern models create relationships among the test points. This algorithm uses spatial information within each of the four quadrant areas (Fig. 4). However, glaucomatous visual field defects occur in a region anatomically corresponding to where optic disc changes occur. Therefore, it would be appropriate to adopt a model that considers glaucomatous structural and functional relationships, such as a glaucoma spatial filter. Rubinstein et al.^11^ compared five different spatial pattern models, which included a model derived from the glaucoma spatial filter^23^, by computer simulation. Their results did not show an obvious advantage of the spatial pattern model using the glaucoma spatial filter, however. Furthermore, the VF test is also an important examination for retinal and neuro-ophthalmic diseases. Therefore, we think that a specific spatial pattern model is not preferable in the clinic for general examinations. Our spatial model could be a reasonable option at present.

The other limitation is the definition of WL. Simulations were run to find an optimal maximum WL value and an attenuation rate in inverse proportion with 20 VFs as preliminary experiments (data not shown). Optimization was achieved with a maximum WL value of 0.25. However, our definition of WL still leaves much to be considered, for instance, differences in individual biometrics^24–26^, calculation methods and dependencies of eye diseases. Further studies are needed to consider a more appropriate WL and to verify whether it is superior to other WLs in clinical practice.

In summary, we developed a new visual field test algorithm that utilizes spatial information. Our results demonstrate that a higher WL between the tested location and neighboring locations could result in fewer test presentations than the non-weighted test. Although using spatial information is beneficial for reducing the test time, the higher fixed WL test has limited accuracy for glaucomatous VFs. The AIZE, in which the WL is adjusted based on spatial relations, is a useful time saving test algorithm for glaucoma patients.

## Methods

### ZEST

AIZE uses the ZEST method, which is based on a maximum likelihood Bayesian technique and was described in previous literature^27^. Briefly, in the ZEST, each test location is associated with a prior probability mass function (PMF), which defines the probability of a given sensitivity threshold. At randomly selected locations, the stimulus intensity equal to the mean of the PMF (prior PMF) is presented.

The patient response (likelihood of seeing a stimulus) is multiplied with the prior PMF according to whether the stimulus was seen or was not seen. A new PMF is generated (posterior PMF), and then a stimulus at the level of the mean value of this newly generated PMF is presented as the next prior PMF. The ZEST repeats those steps until the posterior PMF has satisfied the termination criteria.

### AIZE

**Figure 5:**
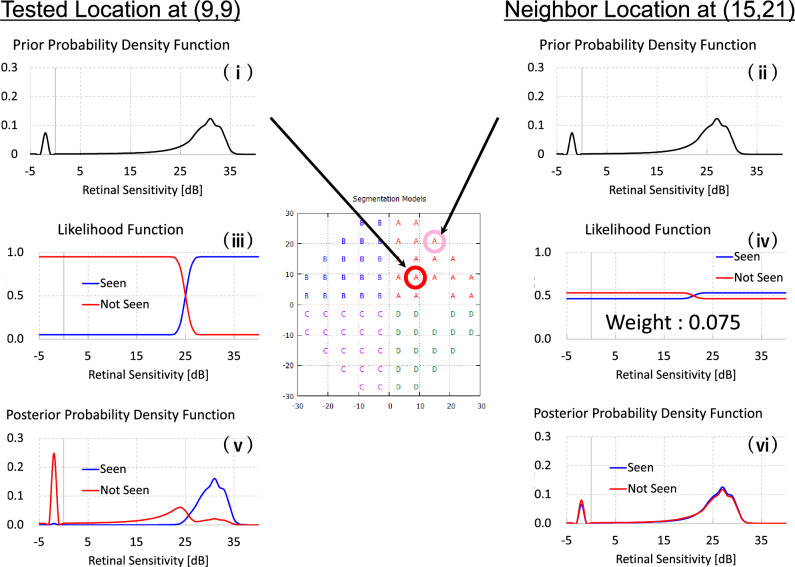
Example of visual field test step with AIZE. A prior PMF at (9,9) location (1) and (15,21) location (2). A stimulus is presented at (9,9) location, the prior-PMF is multiplied by a likelihood function (seen: red, not seen: blue) (3) and the prior PMF at (15,21) location is multiplied by a weighted likelihood which varies according to a distance from the tested location (4). A posterior PMF at (9,9) location is generated (5) and the PMF at (15,21) location is updated simultaneously (6). PMFs of other test locations in the A area are updated with the same manner.

The initial PMF for the visual field test locations is generated by longitudinal glaucomatous VF data^28,29^ and normal VF data tested by the imo perimeter^20^ (CREWT Medical Systems, Inc., Tokyo, Japan) (one hundred seventy-one eyes of 171 healthy subjects who underwent VF tests with a 4–2 dB staircase procedure). The data were distributed between glaucoma and normal VFs in a 1:1 ratio. The PMF ranges from − 2 to 50 dB, and the shape of the PMF is bimodal. Negative intensities and intensities of 40 or above are not represented in the actual perimetric test; instead, they are used to prevent estimated visual sensitivity (mean value of PMF) from tending to extreme values in the process of the thresholding test. While the test procedure for the AIZE is nearly identical to that of the ZEST, the AIZE also uses spatial information for estimating visual sensitivities. The VF is segmented into four areas (Fig. 4). AIZE starts to test an initial location that is selected randomly and updates not only the PMF of the tested location but also the PMFs of neighboring locations in accordance with the patient’s response. The first fifty stimulus are randomly selected from the test locations within fifteen degrees regardless of the quadrant. All subsequent test locations are chosen from the whole test locations randomly. This process continues until all test locations have reached the termination criteria. The strength of the spatial relation between the tested location and neighboring locations is defined as the weighted likelihood (WL),

$$ {\text{WL}} = {1}/(0.{5} \times d + {4}) $$

where *d* (degree) is the distance from the test point. Locations closer to the tested point have a stronger effect on the PMF shape if the affected area of the AIZE WL is within the same quadrant (Fig. 5). The AIZE then computes the variance σ of the posterior PMF for each test point.

$$ \sigma = \left( { - 0.{1} \times {\text{dB}} + {4}.{8}} \right) \times \left( {{1} + {\text{r}}/{3}0 \times 0.{2}} \right) $$

where dB and r represent the brightness of the stimulus indicator (dB) and the distance from the center (degree), respectively.

The AIZE terminates if (1) the variance of the PMF reaches a value predetermined by a function of visual sensitivity and eccentricity. The variance (σ) is calculated by the formula described above and the terminal value ranges from 2.0 to 5.8, or (2) the maximum number of stimuli presented at one location is five. When a test location doesn’t reach a value of the terminal variance within five test times, the estimated sensitivity is determined by the mean value of last PMF. Additionally, a test location where doesn’t reach a value of the terminal variance within 5 test times is tested once or twice again for verification if the estimated sensitivity is less than 15 dB. (Preparatory simulation confirmed that five is an optimal maximum limit to simultaneously shorten the test time and secure reliability. Our preparatory data also showed that the fluctuation of test–retest was larger from 7 to15 dB. We simulated VF tests as a cut off-value with 7, 9, 11, 13, 15 and 17 dB. The 15 dB was better balanced results (total number of presentations and test–retest variability) among them. Therefore, we determined the 15 dB as a cut-off value to proceed the verification test.). With regard to once or twice verification test, the verification test is done once when a patient doesn’t respond the stimuli less than 15 dB, while the twice tests are carried out when a patient responds the stimuli less than15 dB. In the former case, the estimated sensitivity is determined without taking into account the verification test response. On the other, the verification test responses (responses of twice tests) are included to estimate the sensitivity in the latter case.

The AIZE ends when every test location meets either of the above (1) and (2) conditions.

### Computer simulation

**Figure 6:**
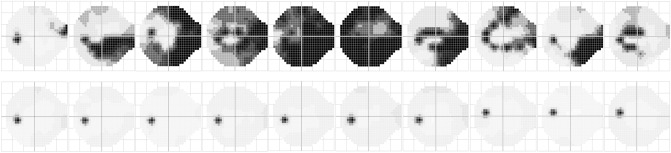
Input VF data (upper). Glaucoma: median MD was − 10.55 dB, range − 0.40 to − 20.82 dB (lower). Normal: median MD was − 0.77 dB, range − 1.78 to 1.50 dB.

The test procedure was implemented by computer simulation until all test locations reached the termination criteria. Patient responses to the stimuli were modeled based on frequency-of-seeing curves with predetermined rates of false-positive (FP) and false-negative (FN) responses. Five error conditions for the frequency-of-seeing curves were simulated: (3% FP, 3% FNs), (9% FP, 9% FN), (15% FP, 15% FN), (15% FP, 3% FN), and (3% FP, 15% FN). The slope of the frequency-of-seeing curve varies with visual sensitivity and was modeled as the standard deviation of the cumulative Gaussian distribution function^30^. The maximum standard deviation for the slope was allowed for 4 dB. We used twenty sets of empirical VF data produced by the 24–2 SITA-standard test on the HFA, 10 glaucomatous VFs [mean deviation (MD): median − 10.55 dB, range − 0.40 to − 20.82 dB] and 10 normal VFs (MD: median − 0.77 dB, range − 1.78 to 1.50 dB) (Fig. 6), as input VF data. Simulations were run 1000 times on each of the 5 error conditions for the 10 glaucomatous and 10 normal VFs; thus, the total number of trials was 100,000.

The data collection was performed in accordance with the Helsinki Declaration, and this study was approved by the Ethics Committee of the Kindai Faculty of Medicine. All patients were given detailed study information, written and oral, and gave informed consent for participation.

### Analysis

The total number of test presentations was counted to assess the time saving capability of the tests. The accuracy of the test algorithm was evaluated by the root mean square error (RMSE) between the true (input) sensitivity and the estimated threshold value. The WL of the AIZE varies as a function of the distance from the tested location. To validate the performance of the AIZE, the non-weighted (WL = 0) and fixed-weighted (WL = 0.33) test algorithms were also simulated under the five FP and FN error conditions. The Wilcoxon signed-rank test was used to compare the RMSE and the total number of test presentations required at all test locations among the AIZE, the non-weighted test and the fixed-weighted test. Additionally, we counted the number of test locations classified by the RMSE values to evaluate test–retest variability at the same test location.

## Data Availability

All relevant data are within the paper. Further data supporting the findings of this study are available from the corresponding author, upon reasonable request.
